# A Simple Way to Estimate a Difficult Sleeve Gastrectomy Prior to Operating

**DOI:** 10.1007/s11695-024-07093-9

**Published:** 2024-02-19

**Authors:** Yalcin Burak Kara, Halil Buluc, Mehmet Elgezen, Edanur Yildiz, Samet Yardimci

**Affiliations:** 1 General Surgery Department, Bahcesehir University VM Medical Park Pendik Hospital, Fevzi Çakmak Mahallesi, D100, Cemal Gürsel Cd. No: 9, Pendik, 34899 Istanbul, Turkey; 2 Department of Anestesiology, VM Medical Park Pendik Hospital, Fevzi Çakmak Mahallesi, D100, Cemal Gürsel Cd. No: 9, Pendik, 34899 Istanbul, Turkey; 3 Department of Radiology, VM Medical Park Pendik Hospital, Fevzi Çakmak Mahallesi, D100, Cemal Gürsel Cd. No: 9, Pendik, 34899 Istanbul, Turkey; 4 School of Medicine, Bahcesehir University, Sahrayı Cedit, Batman Sk., No: 66, Kadıkoy, 34734 Istanbul, Turkey; 5 General Surgery Department, Istinye University VM Medical Park Pendik Hospital, Fevzi Çakmak Mahallesi, D100, Cemal Gürsel Cd. No: 9, Pendik, 34899 Istanbul, Turkey

**Keywords:** Difficult bariatric surgery, Laparoscopic sleeve gastrectomy, Subcutaneous fat tissue, Total fat tissue

## Abstract

**Background:**

Today, bariatric procedures are common. These surgeries’ difficulties are classified as patient- or surgical team–related and are estimated by body mass index (BMI). More efficient methods are needed to help surgeons. This study evaluated the effect of measuring patients’ subcutaneous fat tissue thickness (SFT) and umbilicus-xiphoid (DXU) to anticipate surgical difficulties.

**Material and Methods:**

This was a prospective retrospective data analysis study. Laparoscopic sleeve gastrectomy patients seen between May and October 2022 were included in the analysis and divided into three groups, according to a surgeon’s assessment. All patients’ SFT, DXU, rectus muscle thickness, total fat tissue amount (TFT), and operational time were recorded prospectively and analyzed.

**Results:**

In all, 151 patients were included in the study; of these, 124 (82.1%) were women and 27 (17.9%) were men. Their mean BMI value was 41.1 ± 6.2. Based on expert’s opinion, we classified three groups: easy (*n* = 123, 81.5%), intermediate (*n* = 22, 14.6%), or difficult (*n* = 6, 4%). When the easy group was compared to the intermediate/difficult groups, we found that intermediate/difficult groups’ SFT values were statistically significantly higher than the easy group (*p* = 0.000). Also, the intermediate/difficult group’s TFT value was statistically significantly higher than the easy group (*p* = 0.000). We found no statistically significant differences between groups’ DXU and rectus muscle thickness.

**Conclusion:**

This is the first study to anticipate sleeve gastrectomy difficulty using SFT and TFT. This is an easy technique to apply and no additional costs. Anticipating difficulties based on these criteria can ensure necessary preparations are made and help avoid complications.

**Graphical Abstract:**

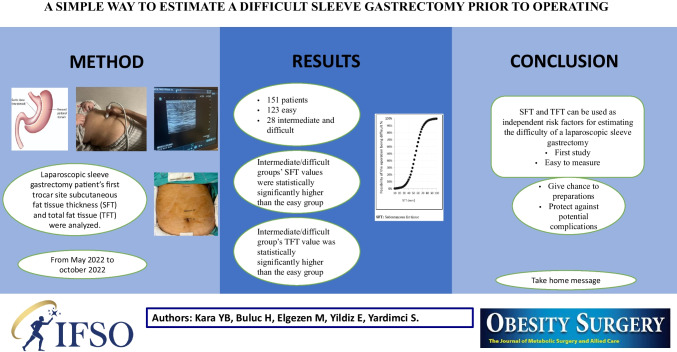

## Introduction

Morbid obesity is a major healthcare problem among all sectors of the world’s population and can be difficult to manage permanently without surgical intervention. Laparoscopic sleeve gastrectomy, which was first described by Hess in 1988 and is a part of the duodenal switch procedure, has grown rapidly in popularity in recent years. It is currently the most commonly performed bariatric operation all over the world [1–4].

The factors that affect the difficulties associated with bariatric surgery can be classified as patient- or surgical team–related ones [5]. Patient-related difficulties include a high body mass index (BMI), weight over 150 kg, a large hiatal hernia, liver cirrhosis, an enlarged liver, a previous instance of pancreatitis, and previous laparotomy. Surgical team–related difficulties include inappropriate trocar replacement and anesthetists or assistants who are less experienced with bariatric procedures [6, 7].

One of the most commonly used parameters to indicate bariatric surgery is BMI. Surgeons often estimate the difficulty of a proposed surgery based on a patient’s high BMI. Patients with a BMI greater than 60 kg/m^2^ are difficult to operate on, while those with a BMI lower than 35 kg/m^2^ are typically easier. The surgical difficulty increases as BMI increases [6, 8].

According to the latest guidelines from the United States’ National Institutes of Health and the American Society for Metabolic and Bariatric Surgery, surgeons can perform bariatric surgeries on the patients with a BMI over 30 kg/m^2^ [9], but they cannot predict pre-operatively the difficulties of surgeries performed on patients with a BMI level between 30 and 60 kg/m^2^.

If a simple determination can be made pre-operatively about the ease or difficulty of the procedure, the chances for surgical success will increase, as the care team can make preparations such as implementing a pre-operative liver size–reducing diet, avoiding the planning of concomitant surgeries like cholecystectomies or hernia repairs, and reserving the operating theater to allow for longer operation times [10].

The effect of subcutaneous fat tissue thickness (SFT) and the distance between the xiphoid and umbilicus (DXU) can be measured easily during the pre-operative phase of a laparoscopic sleeve gastrectomy. To the best of our knowledge, the effect of these variables on the difficulty of sleeve gastrectomy has not yet been examined in the literature. The aim of the present study is to evaluate patients’ SFT and DXU, to determine the potential difficulties of laparoscopic sleeve gastrectomy surgery.

## Materials and Methods

This study was designed as an observational prospective cohort study and all data was analyzed retrospectively. Consecutive patients between the ages of 14 and 70, and whose BMI were between 30 and 65 kg/m^2^, who attended the study center, and were scheduled for a laparoscopic sleeve gastrectomy were included in the study. Patients over the age of 70, younger than 14, who had a history of previous open upper abdominal surgery (open cholecystectomy; open splenectomy; open stomach-related surgery, especially gastric band), who had undergone another bariatric surgery (revisional surgery, bypass surgery), who had a previous tummy tuck operation, liposuction, and patients whose pre-operative radiological evaluations could not be performed correctly were excluded from the study.

**Fig. 1:**
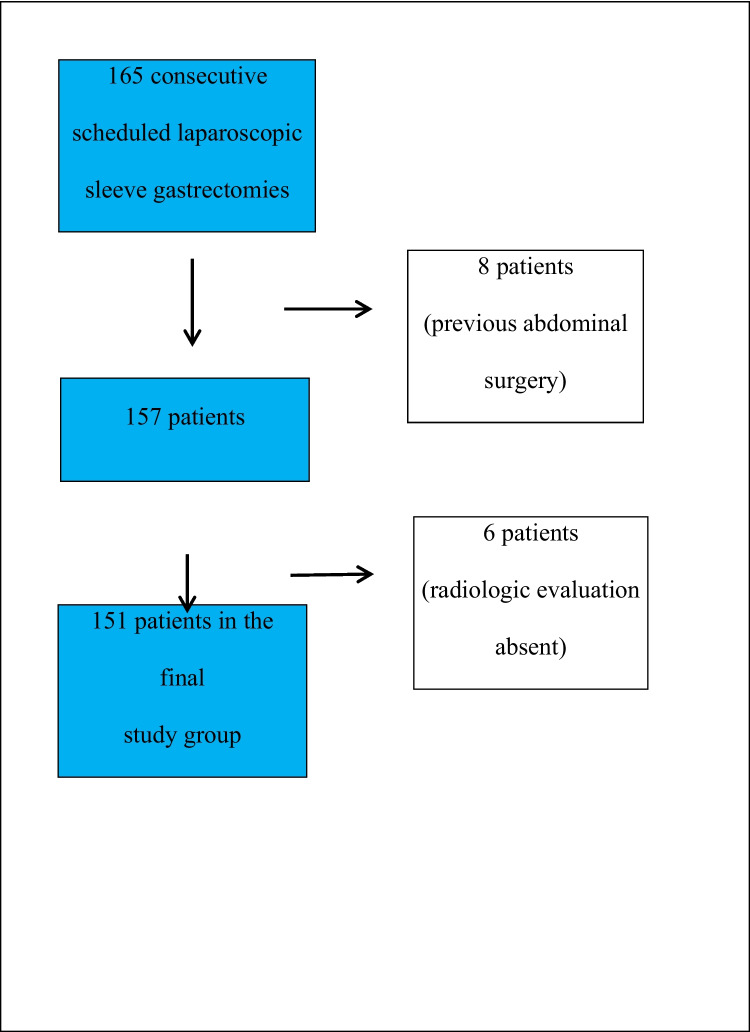
Patient selection and exclusion process

A total of 165 patients scheduled for a laparoscopic sleeve gastrectomy between May and October 2022 were considered for inclusion in the study. Eight patients were later excluded due to their previous surgical history (three had a gastric band, four had a tummy tuck, and one had an open cholecystectomy). Six patients whose radiologic evaluations could not performed correctly were also excluded; 151 patients were ultimately included (Fig. 1).

All patients’ demographic data, such as their age, weight, height, BMI, American Society Anesthesiologists (ASA) score, comorbid diseases, and daily medications were documented. Prior to the operation, all patients were subject to a whole abdomen ultrasonography (USG) by the same radiologist, performed in the center’s radiology unit. The point 15 cm below the xiphoid process was recorded, and the distance between the skin and fascia was measured and recorded as the patient’s SFT via USG. The rectus muscle thickness was also measured by USG and documented. The distance between each patient’s xiphoid process and umbilicus was measured and recorded as their DXU.

All patients were analyzed using a professional body analyzer device (TANITA MC 780). The total fat tissue amount (TFT), amount of abdominal fat tissue, and the percentage of abdominal fat tissue were calculated and documented.

USG and body analysis of all patients were performed in the last 2 weeks period before the surgery.

All operations were performed laparoscopically using the five keyholes technique, as described in the literature [11, 12]. Operational time was calculated for every surgery. Once the distance between xiphoid and umbilicus was measured and marked the 15th cm below of xiphoid process as first trocar site. Then, insufflation succeeded. Time calculations began after the first trocar insertion. After the sleeve gastrectomy specimen was removed from the trocar site, the timing was stopped. The time between these intervals was accepted as the operation time and documented. Thus, trocar site bleeding control and closure time, Nathanson retractor removal time, and drain replacement time (if needed) were excluded from the operational period for all patients as a means of standardization.

All operations were performed by the same surgeon and surgical team. The surgeon who performed the surgeries had a great deal of laparoscopic bariatric surgery experience, more than 1000 cases. He was blinded to the patients’ radiologic evaluations and TANITA body analysis data. At the end of each operation, the surgeon was asked his opinion on the case, whether it had been an easy, intermediate, or difficult operation. All data were collected progressively, and statistical analyses were completed by a professional statistical analysis team. For retrospective studies, study formal consent is not required. Informed consent did not apply.

### The Study Outcomes

Primary outcome of this study is to evaluate to determine the difficulty of laparoscopic sleeve gastrectomy without using BMI value.

Secondary outcome of this study is which parameter significantly affects the difficulty of laparoscopic sleeve gastrectomy as SFT, TFT, DXU, rectus muscle thickness, abdominal fat tissue amount/percentage, gender, and age.

### Statical Analysis

Descriptive statistics of the data, including their mean, standard deviation, median minimum, median maximum, frequency, and ratio values, were used. The distribution of variables was measured with the Kolmogorov–Smirnov test. The independent sample *t*-test and Mann–Whitney *U*-test were used to analyze the quantitative independent data. The chi-square test was used in the analysis of qualitative independent data. Effect levels and cut-off values were investigated with the ROC curve. Cut-off values are determined according to the Youden index. ROC curve, area under the curve gives the highest cut-off value due to the Youden index. The effect level was investigated using univariate and multivariate logistic regressions. The SPSS 28.0 program was used in all analyses.

## Results

Patients’ demographic data are shown in Table 1. These include age, gender, weight, height, BMI, ASA score, surgeon’s (expert) opinion, comorbidities, SFT, DXU, rectus muscle thickness, abdominal fat tissue amount, total fat tissue percentage, abdominal fat tissue percentage, and operational time values.

**Table 1:** Patients’ demographic feature

		Min–max			Median	Mean ± ss/n-%		
Age		14.0	–	70.0	36.0	36.3	±	10.3
Gender	Female					124		82.1%
	Male					27		17.9%
Height		150.0	–	188.0	165.0	165.6	±	7.5
Weight		71.6	–	197.0	110.5	113.3	±	21.5
BMI		30.2	–	63.4	40.5	41.1	±	6.2
ASA score	I					34		22.5%
	II					115		76.2%
	III					2		1.3%
Expert’s opinion	Easy					123		81.5%
	Intermediate					22		14.6%
	Difficult					6		4.0%
Comorbidities	( −)					105		69.5%
	( +)					46		30.5%
Used medications	( −)					103		68.2%
	( +)					48		31.8%
Subcutaneous fat tissue (mm)		13.0	–	72.0	40.0	39.8	±	10.4
Xiphoid-umbilicus (cm)		15.0	–	41.0	22.0	22.8	±	4.0
Rectus thickness (mm)		1.0	–	29.0	14.0	14.4	±	3.7
Total fat tissue amount (kg)		23.2	–	102.7	47.7	50.4	±	13.0
Total fat tissue percentage (%)		28.0	–	63.2	44.1	44.3	±	5.3
Abdominal fat tissue percentage (%)		24.0	–	67.9	50.7	50.3	±	7.8
Operation time (mins)		22.0	–	72.0	45.0	44.1	±	9.1

*Min–Max* minimum–maximum, *BMI* body mass index, *mm* millimeter, *cm* centimeter, *kg* kilogram, *min* minute

The patients’ median BMI value was 40.5 kg/cm^2^, and the median operational time was 45 min. According to the expert’s opinion, 123 cases were easy, 22 cases were intermediate, and 6 were difficult. With this in mind, patients were sorted into one of two groups, easy (*n* = 123) or intermediate-difficult (*n* = 28).

The results of the expert’s opinion concerning the two groups’ members are shown in Table 2. According to these, gender, weight, height, BMI, SFT, and total fat tissue amount were deemed to be significant factors for predicting the difficult operations. The operation time was significantly longer for members of the intermediate-difficult group, compared to the easy group. There were no significant differences between groups’ ages, ASA scores, comorbidities, medications, DXU, rectus muscle thickness, total fat tissue percentage, or abdominal fat tissue percentage (*p* > 0.05) (Table 2).

**Table 2:** Comparison of characteristics between easy and intermediate-difficult group

			Expert’s opinion													*p*-value				
			Easy					Intermediate-difficult												
			Mean ± ss/n-%		Median			Mean ± ss/n-%					Median							
Age		37.0	±	10.5		37.0		33.1	±		8.8			33.0			0.086		^m^	
Gender	Female	106		86.2%				18			64.3%						***0.006***		^X2^	
	Male	17		13.8%				10			35.7%									
Height		164.7	±	7.3		164.0		169.6	±		7.1			170.0			***0.000***		^m^	
Weight		109.7	±	20.7		105.0		129.1	±		17.7			128.0			***0.000***		^m^	
BMI		40.2	±	6.0		39.9		44.9	±		5.3			45.6			***0.000***		^m^	
ASA score	I	30		24.4%				4			14.3%						0.248		^X2^	
	II	91		74.0%				24				85.7%								
	III	2		1.6%				0				0.0%								
Comorbidities	( −)	86		69.9%				19				67.9%						0.831		^X2^
	( +)	37		30.1%				9				32.1%								
Medications	( −)	87		70.7%				16				57.1%						0.163		^X2^
	( +)	36		29.3%				12				42.9%								
Subcutaneous fat tissue (mm)		37.6	±	9.1			36.0	49.3		±		10.2			50.5			***0.000***		^m^
Xiphoid-umbilicus (cm)		22.4	±	3.7			22.0	24.2		±		4.7			23.0			0.065		^m^
Rectus thickness (mm)		14.3	±	3.4			14.0	15.3		±		5.0			15.0			0.219		^m^
Total fat tissue amount (kg)		48.6	±	12.7			45.8	58.9		±		11.3			58.1			***0.000***		^m^
Total fat tissue perc		44.1	±	5.2			43.7	45.6		±		5.7			46.2			0.188		^t^
Abdominal fat tissue perc		49.9	±	8.0			50.5	52.5		±		6.3			51.0			0.160		^m^
Operation time (mins)		42.0	±	7.7			42.0	53.7		±		8.3			53.0			***0.000***		^t^
Operation Time	< 50	96		78.0%				8				28.6%						***0.000***		^X2^
	≥ 50	27		22.0%				20				71.4%								

Bold italic means statistically significant

^t^Independent sample *t*-test/^m^Mann-Whitney *U*-test/^X2^chi-square test

*BMI* body mass index, *ASA* American Society of Anesthesiologists, *Perc* percentage

The results of the univariate and the multivariate logistic regression analyses for the factors influencing the difficulty level of the surgeries are shown in Table 3. In the univariate model, we observed a significant efficiency in differentiating the patients in the easy group from the intermediate-difficult group by gender, height, weight, BMI, SFT, and TFT (*p* < 0.05). In the multi-variate model, a significant independent efficiency of skin thickness and male gender were observed when sorting patients into the easy and intermediate-difficult groups (*p* < 0.05).

**Table 3:** Univariate and multivariate analyses of factors influencing operations’ difficulty

	Univariate model					Multivariate model				
	OR	%95 CI*			*p*-value	OR	%95 CI*			*p*-value
Gender	3.464	1.371	–	8.755	***0.009***	3.687	1.176	–	11.559	***0.025***
Height	1.091	1.031	–	1.154	***0.002***					
Weight	1.041	1.020	–	1.063	***0.000***					
BMI	1.128	1.052	–	1.209	***0.001***					
SFT	1.137	1.077	–	1.201	***0.000***	1.172	1.097	–	1.252	***0.000***
Total fat tissue Amount	1.059	1.025	–	1.095	***0.001***					

Bold italic means statistically significant

Logistic regression (forward LR)/CI* confidence interval

*BMI* body mass index, *SFT* subcutaneous fat tissue

We were surprised to find that BMI and weight were not significantly different in the multivariate analyses (Table 3).

**Fig. 2:**
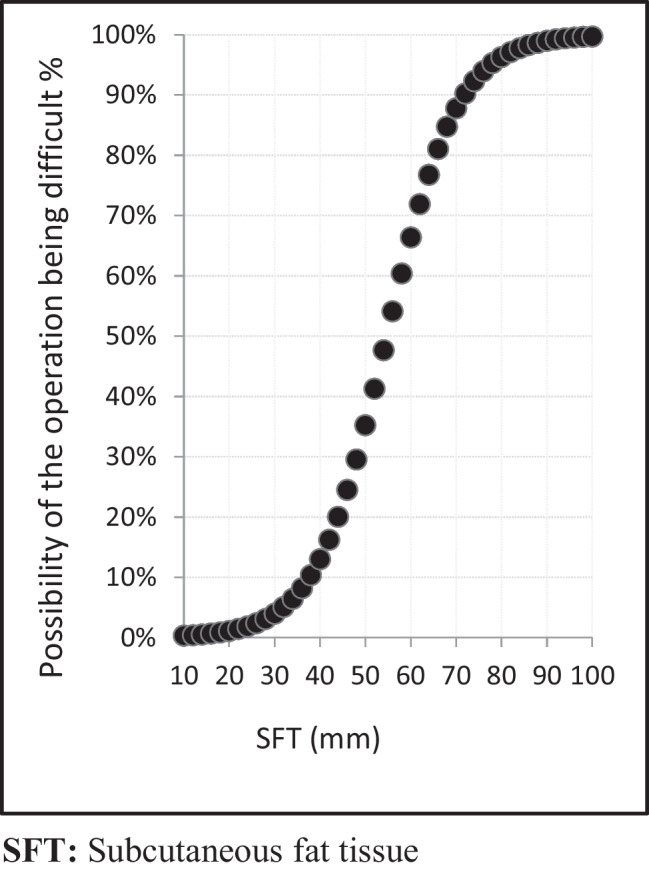
Relationship between difficulty of the operation and subcutaneous fat tissue

**Fig. 3:**
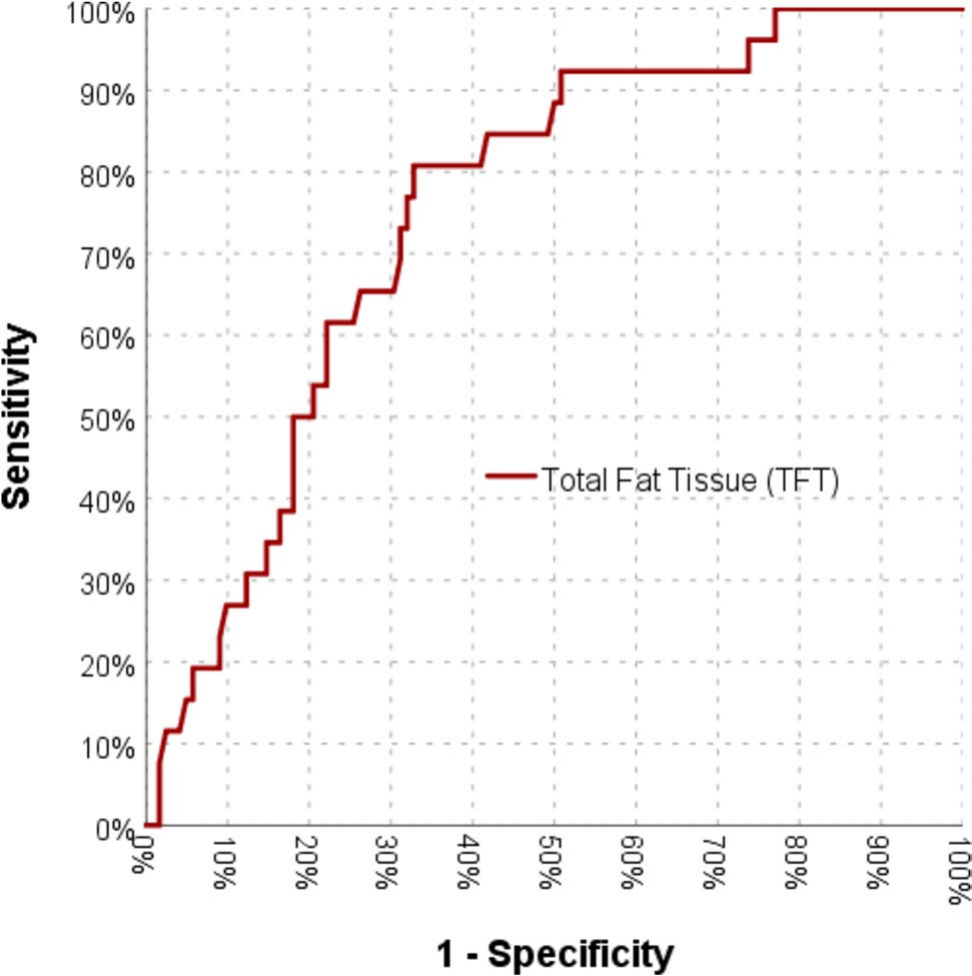
Relationship between difficulty of the operation and total fat tissue

The results of SFT’s and TFT’s effect on the difficulty of sleeve gastrectomy procedures are depicted in Fig. 2 and Fig. 3. The critical value of SFT and TFT are shown in Table 4.

**Table 4:** Critical values of Subcutaneous fat tissue and total fat tissue

		Easy	IM/D	Sensitivity	Pozitive Est	Specifity	Negative Est
SFT(mm)	≤ 40	**80**	4	85.7%	35.8%	65.0%	95.2%
	> 40	43	**24**				
TFT (kg)	≤ 50.8	**82**	5	80.8%	34.4%	67.2%	94.3%
	> 50.8	40	**21**				

*SFT* subcutaneous fat tissue, *TFT* total fat tissue, *Est* estimation, *IM/D* intermediate/difficult

### Complications

There was no leakage of laparoscopic sleeve gastrectomy (0/151). The bleeding rate after surgery was 0.013 (2/151). One of these two patients did not need any surgical intervention, as a blood transfusion alone stopped the bleeding. The other patient underwent a diagnostic laparoscopy, but no active bleeding was observed; the abdomen was cleaned and drainage was initiated. No trocar site hernia was observed. There were no instances of 30-day mortality or readmission.

## Discussion

While many laparoscopic sleeve gastrectomy procedures have been performed all over the world, surgeons remain unaware of the challenges they may face during the next sleeve surgery they perform. Traditionally, a patient’s BMI value has been the most frequently used parameter for anticipating surgical difficulties, but sometimes this value alone is insufficient.

If we can predict when a surgery may be challenging or difficult based on simple measurements taken before the surgery, we can achieve safer surgeries and lower complication rates by taking necessary precautions. This would also be beneficial for surgical training, so that surgeons who are in early stages of the learning curve can benefit immediately and be taught how to address potentially difficult and challenging cases.

In our surgical practice, we sometimes noticed cases that were incompatible with the difficulty of high BMI (over 50 kg/m^2^) and last shorter time than expected. When we examined these cases, we encountered that SFT value was shorter and DXU value was longer which led to the design of the study.

The aim of this study was to evaluate predictions about challenging or difficult cases of sleeve gastrectomy, regardless of BMI value. SFT, DXU, abdominal fat tissue amount, and percentage may affect both surgery times and the surgeon’s pre-operative opinion.

Most bariatric surgeons believe a sleeve surgery will be challenging if the patient’s BMI is greater than 60 kg/cm^2^. Nguyen et al., Artuso et al., and Major et al. each noted in their studies that a BMI over 60 kg/cm^2^ could lead to challenges related to surgical time. In our study, the increase of BMI significantly influenced the technical difficulties of the surgeries; this was a finding in line with the literature [5, 7, 8].

The surgery of patients with the same BMI sometimes not have similar difficulty, which forces us to look forward for other values other than BMI in assessing difficulty. In addition to BMI value, SFT, total fat tissue amount, and gender were found to have a significant effect on whether or not a sleeve surgery was deemed “challenging” in our study (*p* < 0.05) (Table 3). A search of the literature revealed no related study that evaluated the relationship between how challenging a sleeve surgery was and patients’ SFT or total fat tissue. This is the first study, to our knowledge, that has measured these two values as a means of estimating the difficulty of a sleeve gastrectomy. SFT, which is measured by ultrasound, was found to be an independent factor for estimating the difficulty of a sleeve gastrectomy, according to our expert’s opinion (Table 3). We also detected a cut-off value for SFT; patients with an SFT greater than 40 mm tended to have statically significant difficult laparoscopic sleeve surgeries (Table 4). As SFT value increases, the difficulty of surgery also increases; we can explain this by the fact that a high SFT value limits the mobility of surgeons’ hands and prevents them from performing the necessary fine skill movements. Another factor is the narrowing of the abdominal field of vision.

SFT can easily be used in clinical practice. Because pre-operative ultrasound is a routine part of the bariatric patient’s preparation protocol, this technique does not lead to a loss of time or incur additional costs. However, the radiologist must be informed about measuring subcutaneous fat tissue at the level of the first trocar insertion area. If the measurement is greater than 40 mm, special preparations should be made, as the surgery will likely become difficult.

This study is also the first to use SFT as a parameter for differentiating difficulties noted in the literature. Tałałaj et al. studied body fat tissue amounts, abdominal fat tissue percentages, and body fat tissue percentages based on dual-energy X-ray absorptiometry [13]. Sun et al. studied the abdominal fat tissue amount referred to as the “subcutaneous abdominal fat index” using MRI [14]. In our study, these values were obtained by TANITA. This measurement method does not cause time loss or additional cost, as USG does, as it is routinely applied during bariatric surgery preparations. In our study, the total fat tissue amount was found to be an independent factor for anticipating a difficult sleeve gastrectomy, according to our expert’s opinion (Table 3). This finding can easily be used in clinical practice as well.

Dugan et al. suggested that males have higher risk rates than females, when considering major surgical complications like bleeding, leakage, and death [15]. When we studied the gender effect on difficult sleeve surgery, we noted that 86.2% of the easy group were women, while the proportion of women in the intermediate-difficult group was 64.3%. Both univariate and multivariate analyses showed that male gender caused statically significant difficult sleeve gastrectomies in our study (*p* < 0.05). These findings are compatible with the literature and Dugan et al.’s explanation that males with obesity tend to have more centralized weight, with thick abdominal walls and more intraabdominal fat tissue, compared to females. In our study when we analyzed the rectus muscle thickness and abdominal fat tissue percentage, there was no statistical difference between the two groups.

Katkhouda et al. and Clapp et al. [16, 17] studied the port placement choices in bariatric surgery and suggested that the landmark area should be the xiphoid process. The first trocar placement area should be 15–18 cm below the xiphoid process. In our study, the first trocar site was used, 15 cm below the xiphoid process, as reported in the literature. We also studied the distance between the xiphoid and umbilicus to help predict the difficulties of sleeve surgery. We investigated the hypothesis that a greater distance between the umbilicus and xiphoid would increase the field of view in the upper abdomen and facilitate the surgery, but we did not find a significant difference between the two groups in this evaluation.

In addition to the above, we investigated the hypothesis that trocar movements and laparoscopic instrument movements would be more limited in patients with greater rectus muscle thickness. Kim et al. and Tokumoto et al. studied rectus abdominis muscle thickness by using CT scan [18, 19], while the measurement was investigated via USG in our study. The area 15 cm below the xiphoid process was identified as the target point area for USG to measure rectus muscle thickness. We found no statistically significant relationship between the laparoscopic sleeve surgery difficulty level and rectus muscle thickness.

Karip et al. observed that previous surgical history, and especially abdominoplasty, could lead to difficulties in bariatric surgery. In our study, eight patients were excluded because of previous surgical history; we made this decision to avoid bias when comparing patients [20].

In a search of the literature, we found that many studies suggested that laparoscopic sleeve gastrectomy had acceptable safety risk and was an appropriate procedure, compared to other bariatric surgical operations. When comparisons were made of short-term postoperative complications, such as readmission, re-intervention, re-operation, unplanned intensive care unit stays, and the 30-day mortality rate, laparoscopic gastric bypass had a two to threefold greater potential risk rate than laparoscopic sleeve gastrectomy in both adult and pediatric populations [21–24]. For this reason, among patients known to be of high risk, laparoscopic sleeve gastrectomy should be the first choice when planning a bariatric surgery.

In our study, a SFT above 40 mm, total fat tissue greater than 50.8 kg, and male gender had statically significant effects on the surgeon’s opinion of the surgery’s level of difficulty and the time needed for surgery. Estimating a difficult case before the operation has several advantages. For instance, in these patients, gastric bypass should not be the first operational choice. These patients’ operations might also benefit from being scheduled as the first case of the day, as the needed operational time can be planned for longer than routinely scheduled. With potential complications in mind, the number of cases on the operational calendar of the day may also be arranged more accurately. Further, patients with these features might be placed on the operational calendar on the different days, to help promote the success and comfort of the surgeon and surgical team. A second and/or more experienced surgeon could also be scheduled for these potentially difficult patients. Longer trocars and laparoscopic instruments may be prepared before these surgeries.

It can be useful for single-day discharge program or the clinics which try to use fast-track surgeries protocols.

This parameter can also be used for educational programs in a university hospital to decide the first case of young surgeons. Early-career surgeons, or those who are at the beginning of bariatric surgery learning curve and find themselves trying to perform these difficult cases, may also lead to various complications or even discourage the surgeon. Furthermore, anticipating the difficulty of an operation is important for the surgeon’s comfort and any case-based anxiety he or she might experience. This is as important to consider as any other surgical complication.

This study has some limitations. Although the number of patients in the study was 151, the intermediate group consisted of 22 patients, and difficult group consisted of 6 patients. If there was a larger sample, the number of patients in difficult group would, presumably, be greater. However, all operations were performed by a single experienced surgeon. If the study had been a multicenter one or included multiple experienced surgeons, the data would be more heterogeneous because of the different experiences of different surgeons. These, in turn, could lead to standardization problems. If the number of surgeons was more than one, it could cause different operational times by different surgeons and surgical time cannot be used as a parameter in this study. The study focused on the work of only a single surgeon, and those surgeries expected to be “difficult” took longer in our study. This implies that the study, which included some subjective criteria as “difficulty,” was still supported by objective data and the duration of surgeries is compatible with surgeon’s decision on difficulty in statical analysis.

Single-surgeon designed study also creates a limitation to our study for applicability to large community. The study should be supported large sample and single-surgeon designed other studies. another limitation is that when all surgical operations were performed by one surgeon, the time of surgery (first case or last case) could cause to change the difficulty level. In our study, we overcame to try this problem by a surgeon with experience of over 1000 cases.

## Conclusions

This is the first study, to our knowledge, to determine that SFT and TFT are independent factors for estimating the difficulty of a laparoscopic sleeve gastrectomy. These two data points can be determined via USG and TANITA measurements during routine pre-operative preparations without wasting time or incurring extra costs, and the SFT and TFT can be used akin to a BMI value to determine the potential difficulty of a laparoscopic sleeve gastrectomy. Predicting that a surgery may be difficult, and making the necessary preparations for the same, can be prepared in advance against potential complications.
